# Lung Cancer in Never-Smokers: Risk Factors, Driver Mutations, and Therapeutic Advances

**DOI:** 10.3390/diagnostics16020245

**Published:** 2026-01-12

**Authors:** Po-Ming Chen, Yu-Han Huang, Chia-Ying Li

**Affiliations:** 1 Research Assistant Center, Show Chwan Memorial Hospital, Changhua 500, Taiwan; rabbitshuyaoming9@gmail.com; 2 Department of Nursing, Central Taiwan University of Science and Technology, Taichung 406, Taiwan; 3 Division of Thoracic Surgery, Department of Surgery, Show Chwan Memorial Hospital, No. 542, Section 1, Zhongshan Road, Changhua 500, Taiwan; necotako@gmail.com

**Keywords:** lung cancer, never-smokers, driver mutations, air pollution, PM_2.5_, targeted therapy, immunotherapy

## Abstract

**Background and Objectives**: Lung cancer in never-smokers (LCINS) has become a major global health concern, ranking as the fifth leading cause of cancer-related mortality. Unlike smoking-related lung cancer, LCINS arises from complex interactions between environmental carcinogens and distinct genomic alterations. This review summarizes current evidence on environmental risks, molecular features, and therapeutic progress shaping lung cancer management. **Methods**: A narrative review was conducted to examine risk factors for lung cancer in non-smokers. Studies reporting driver mutations in never-smokers and smokers were identified across major lung cancer histological subtypes, including small-cell lung cancer (SCLC), lung adenocarcinoma (LUAD), squamous cell carcinoma (SCC), and large-cell carcinoma (LCC). In addition, PubMed was searched for phase III trials and studies on targeted therapies related to driver mutations published between 2016 and 2025. **Results**: Environmental factors such as cooking oil fumes, radon, asbestos, arsenic, and fine particulate matter (PM_2.5_) are strongly associated with LCINS through oxidative stress, DNA damage, and chronic inflammation. *EGFR*, *PIK3CA*, *OS9*, *MET*, and *STK11* mutations are characteristic of never-smokers, in contrast to *TP53* mutations, which are more common in smokers. Recent advances in targeted therapy and immunotherapy have improved survival and quality of life, emphasizing the importance of molecular profiling for treatment selection. **Conclusions**: LCINS represents a distinct clinical and molecular entity shaped by complex interactions between environmental exposures and genetic susceptibility. Genetic alterations promote tumor immune evasion, facilitating cancer development and progression. Continued advances in air quality control, molecular diagnostics, and precision therapies are essential for prevention, early detection, and reduction of the global disease burden.

## 1. Introduction

In the general population, lung cancer remains the leading cause of cancer-related mortality worldwide, with incidence and mortality patterns strongly influenced by smoking prevalence, sex, and geographic region [1]. In contrast to smoking-related lung cancer, lung cancer in never-smokers (LCINS) represents a distinct epidemiologic entity with unique risk factors and clinical characteristics [2]. LCINS accounts for a substantial proportion of lung cancer cases globally, with reported estimates varying by region, particularly in East Asia, where incidence among women is comparatively higher [2]. LCINS represents a distinct clinical and epidemiologic entity, accounting for a substantial proportion of lung cancer cases worldwide, with marked geographic variability influenced by environmental and lifestyle factors [3]. Lung adenocarcinoma is the predominant histologic subtype in never-smokers, whereas squamous cell carcinoma and small-cell lung cancer are more frequently observed in smokers [3]. Histologic patterns differ substantially between smokers and never-smokers, reflecting distinct etiologic mechanisms, exposure profiles, and molecular characteristics [4].

Tobacco smoking is the most established and powerful risk factor for lung cancer worldwide [5]. Increasing evidence also highlights the important roles of non-tobacco determinants such as air pollution, occupational exposures, and various environmental and socioeconomic factors in shaping the global lung cancer burden [6]. Recent studies suggest possible associations between lung cancer risk and factors such as ambient temperature, urbanization, and cooking practices, although these relationships remain incompletely understood and show variability across populations and study designs [6]. Smoking remains the dominant contributor to lung cancer, yet rapid urbanization, climate variability, and lifestyle transitions appear to be influencing disease patterns and prevalence [7].

**Figure 1:**
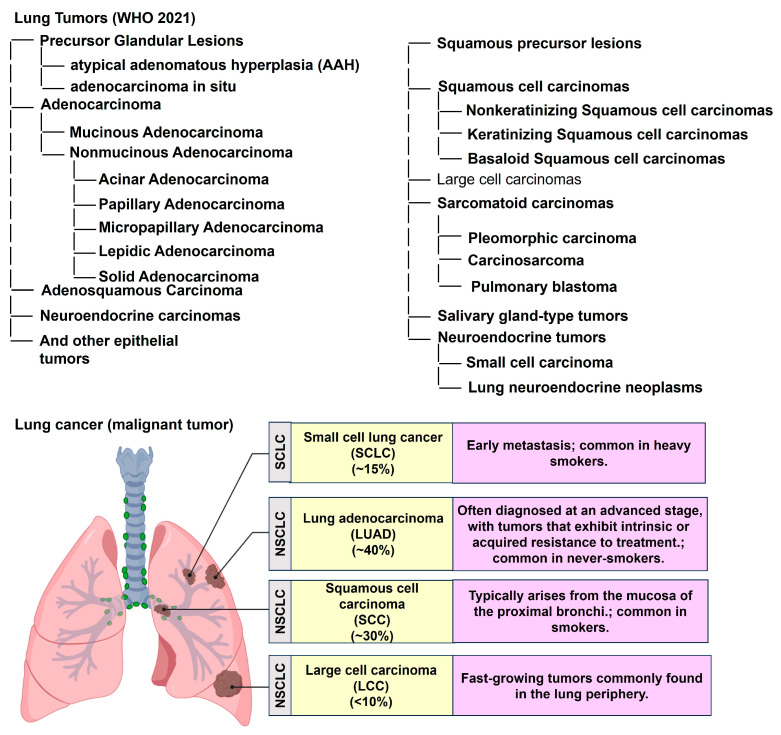
Classification and distribution of primary malignant lung tumors according to the World Health Organization (WHO) 2021 guidelines. NSCLC: Non-small-cell lung cancer. Icons were created with BioRender.com.

**Figure 2:**
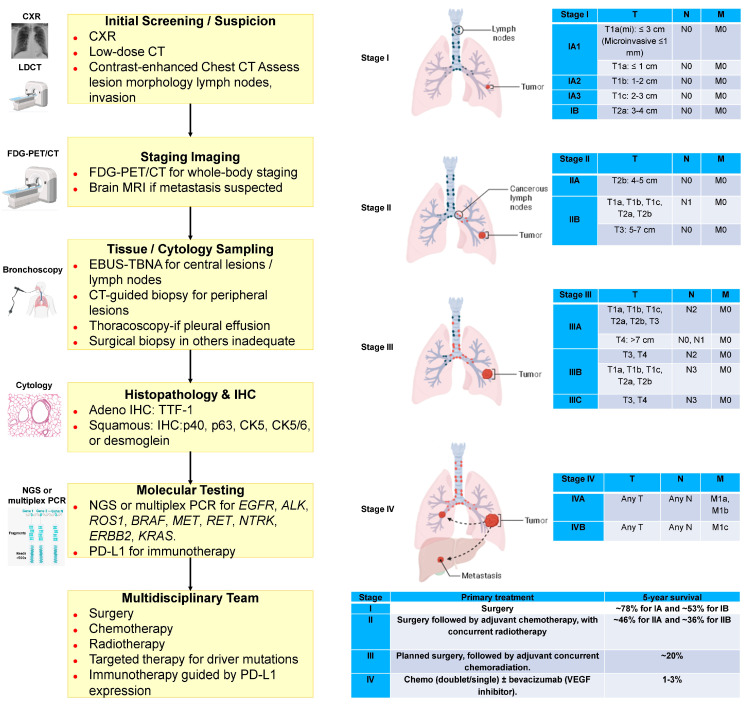
Diagnostic workflow and TNM classification in lung cancer. T: Primary tumor. Features. N: Metastases in the surrounding lymph nodes. M: Distant metastases. N1: Metastases in ipsilateral peribronchial, hilar, or intrapulmonary lymph nodes. N2: Metastases in ipsilateral mediastinal or subcarinal lymph nodes. N3: Metastases in contralateral mediastinal, hilar, or supraclavicular lymph nodes. M1a: Contralateral lung, pleural, or pericardial metastases. M1b: Single distant organ metastasis. M1c: Multiple or multi-organ metastases. CXR: chest X-ray. FDG-PET/CT: 18F-fluorodeoxyglucose positron emission tomography/computed tomography. EBUS-TBNA: Endobronchial ultrasound-guided transbronchial needle aspiration. IHC: Immunohistochemistry. NGS: Next-generation sequencing. PCR: Polymerase chain reaction. Icons were created with BioRender.com.

The classification and distribution of primary malignant lung tumors, based on the World Health Organization (WHO) 2021 guidelines, are summarized in Figure 1 (Icons were created using BioRender.com). Lung cancer continues to account for a major share of the global cancer burden, with an estimated 2.5 million new cases (12.4% of all cancers) and 1.8 million deaths (18.7% of all cancer deaths) in 2022 [8]. It is the most common cancer in men and the second most common in women, with a global male-to-female ratio of about 2 [8]. This ratio varies regionally, from near parity in North America and Northern Europe to four- to five-fold higher in Northern Africa and Eastern Europe [8]. Lung cancer remains the leading cause of cancer-related deaths globally, with a 5-year survival rate of only 10–20% [9,10]. Most cases are classified as non-small-cell lung cancer (NSCLC, approximately 80%) or small-cell lung cancer (SCLC, approximately 15%). Large cell lung cancer (LCLC), a subtype of NSCLC, accounts for about 9% of all lung cancer cases and is often characterized by poor differentiation and an unfavorable prognosis [11,12]. Tobacco use, especially cigarette smoking, accounts for more than 80–90% of all lung cancer cases [11]. The diagnostic workflow for lung cancer, combined with the TNM classification system, offers an objective framework for evaluating tumor burden and guiding therapeutic decision-making [13,14,15] (Figure 2). (Icons were created using BioRender.com.)

Taiwan initiated a national lung cancer early detection program in 2022, offering biennial low-dose CT (LDCT) scans to never-smokers aged 45–74 years for females and 50–74 years for males with a family history of lung cancer [16]. This program was informed by a study that conducted LDCT screening in 12,011 asymptomatic Taiwanese never-smokers aged 55–75 years with risk factors such as having a first-degree relative with lung cancer, exposure to secondhand smoke, or cooking without adequate ventilation. The study reported that 2.7% of individuals with a family history of lung cancer were diagnosed with incident lung cancer, compared with 1.6% among those without a family history (*p* < 0.0001), and that 77.4% of detected lung cancers were diagnosed at Stage I [16].

In LCINS, environmental and lifestyle-related exposures, including air pollution and indoor combustion, are considered major risk factors, whereas tobacco smoking remains the dominant determinant in the general lung cancer population [17]. Environmental exposures, including ambient air pollution, indoor combustion from solid fuels, and occupational exposures, contribute to lung cancer risk in never-smokers, with their relative impact varying significantly by region and socioeconomic context [18]. Current evidence supports a gene–environment interaction model rather than a single dominant mechanism. Environmental carcinogens, including PM_2.5_, cooking fumes, and radon, may induce somatic alterations such as *EGFR* mutations in exons 18–21 [19], gene rearrangements (e.g., *ALK*) [20], and gene fusions (e.g., *RET*) [21], and they interact with inherited susceptibility loci, including the 5p15.33 region [22], which is associated with lung cancer risk in never-smokers and implicates pathways involved in cell cycle control, DNA damage response, immune regulation, and genomic stability.

*EGFR* mutations are the most common driver in LCINS, occurring in 52–74% of non-smoking East Asian women with LUAD, versus ~10% in smoking-related LUAD [23]. Canonical exon 19 deletions and L858R point mutations account for >85% of *EGFR* alterations, with exon 19 deletions more frequent in younger patients and L858R in older patients [24]. The likelihood of *EGFR* mutation decreases with smoking exposure but increases with the number of smoke-free years [25,26]. Uncommon *EGFR* mutations, including exon 20 insertions and rare fusions (e.g., *EGFR*–*RAD51*), are more frequent in never-smokers and are targetable with *EGFR* TKIs [27]. *ALK* rearrangements are the second most common driver in LCINS, present in up to 14% of cases, mostly in never-smokers [28]. *ALK*-driven tumors are almost exclusively adenocarcinomas and are targetable with ALK inhibitors [29].

*KRAS* mutations dominate smoking-related LUAD but are less frequent (5–15%) in never-smokers, and G12D is the most common variant in never-smokers, whereas G12C predominates in smokers [30]. *KRAS^G12D^*-mutant tumors in never/light smokers show lower PD-L1 expression, lower TMB, and poor response to PD-(L)1 monotherapy [31]. *MET* alterations include exon 14 skipping (1–4%) and amplifications (1–6%). *METex14* mutations are enriched in never-smokers and older patients, whereas amplifications are more common in younger patients and may arise as resistance to EGFR/ALK-targeted therapy [29,32]. *MET* fusions (e.g., *KIF5B*–*MET*) occur in never/light smokers and are targetable with crizotinib [33,34,35].

Activating *HER2* mutations occur in 1–5% of NSCLCs, mostly in never-smoking women, predominantly as exon 20 in-frame insertions [36,37]. *HER2* amplifications/overexpression are less clearly actionable, but mutations are sensitive to *HER2*-targeted therapies [38,39]. Additionally, PD-L1 expression is generally low or absent in LCINS and in NSCLCs with non-*KRAS* oncogenic drivers [40,41]. Among *KRAS*-mutant tumors, PD-L1 positivity increases with smoking history, being lowest in never-smokers and highest in current smokers, correlating with pack-years [40]. *METex14*-altered NSCLC may show moderate to high PD-L1, but TMB remains low, and ICI response rates are modest (17–36%) and do not correlate with PD-L1 or TMB. *ALK*- and *ROS1*-rearranged NSCLCs are more frequently PD-L1 positive than EGFR-mutant NSCLCs (70–73% vs. 50%), likely reflecting oncogene-driven PD-L1 upregulation rather than true T cell-mediated immunogenicity predictive of ICI response [42,43,44,45,46,47]. Genetic mutations contribute to tumor immune evasion. Genetic mutations contribute to tumor immune evasion by altering antigen presentation, modulating immune checkpoint signaling, and reshaping the tumor microenvironment, thereby enabling cancer cells to escape immune surveillance and persist despite host antitumor immune responses.

Despite major advances in understanding the molecular and environmental determinants of lung cancer, critical knowledge gaps persist, especially among never-smokers. Elucidating how environmental carcinogens interact with key genomic alterations is essential for improving risk prediction and developing targeted preventive approaches. This review aims to characterize the molecular landscape and therapeutic implications of LCINS. We systematically evaluated histology-specific and smoking-status associated differences in oncogenic driver alterations, focusing on genes more frequently observed in never-smokers, such as *EGFR*, *PIK3CA*, *OS9*, *STK11*, and *MET*. We also integrated these molecular findings with clinical evidence on targeted and immunotherapy strategies, providing a comprehensive overview of precision treatment approaches in this distinct patient population. The integration of molecular diagnostics with novel therapeutic strategies offers new opportunities for precision management of lung cancer in never-smoker populations. This review summarizes current evidence on environmental risks, molecular alterations, and pivotal clinical trials that have transformed modern lung cancer care, with a focus on the distinct biology and treatment landscape of LCINS.

## 2. Materials and Methods

A structured literature search was performed using PubMed. Studies published between January 2016 and October 2025 were considered to capture.

In the first step, studies reporting driver mutation profiles in never-smokers or non-smokers were identified across major lung cancer histological subtypes, including small-cell lung cancer (SCLC), lung adenocarcinoma (LUAD), squamous cell carcinoma (SCC), and large-cell carcinoma (LCC). Eligible studies included large-scale genomic analyses, cohort studies, and molecular epidemiologic investigations that examined mutation frequencies stratified by smoking status and histology. These studies were used to summarize histology-specific and smoking status-associated differences in oncogenic driver alterations. “EGFR,” “PIK3CA,” “OS9,” “STK11,” “MET,” were identified more in never-smoker than Current/Former Smoker.

In the second step, this approach allowed integration of molecular diagnostic findings with clinically relevant treatment evidence. Search terms included combinations of Medical Subject Headings (MeSH) and free-text keywords: “phase III clinical trial,” “lung cancer,” “never-smoker,” “non-smoker,” “small-cell lung cancer,” “lung adenocarcinoma,” “squamous cell carcinoma,” “large-cell carcinoma,” “mutation,” “*EGFR*,” “*PIK3CA*,” ”*OS9*,” “*STK11*,” “*MET*,” “targeted therapy,” and “immunotherapy”. Smoking status was defined based on the information provided in the original studies. Individuals were classified as never-smokers when they were explicitly described as having no history of cigarette smoking. Detailed data regarding smoking quantity, duration, or cumulative exposure (e.g., pack-years) were not consistently reported and therefore could not be assessed. As a result, smoking status in this review was analyzed as a binary variable (never-smoker vs. Current/Former Smoker), and no dose–response or exposure-intensity analyses were performed.

## 3. Results

### 3.1. Characterization of Cooking Oil Fume-Related Carcinogenic Compounds and Their Relevance to Lung Cancer Risk

Cooking oil fumes contain multiple genotoxic and carcinogenic substances implicated in lung carcinogenesis. Prominent among these are polycyclic aromatic hydrocarbons (PAHs) such as benzo[a]pyrene (BaP), which forms through incomplete combustion of organic matter and induces DNA adducts that result in G→T transversions in tumor suppressor genes, including *TP53* and *KRAS* [48,49,50]. Heterocyclic amines (HCAs), generated during high-temperature frying of meat and oil, cause DNA strand breaks and oxidative stress through CYP1A2-mediated metabolic activation [51,52,53]. Volatile nitrosamines, including N-nitrosodimethylamine (NDMA) and N-nitrosopyrrolidine (NPYR), are released when nitrite-contaminated oils are heated and contribute to increased mutagenic burden in airway epithelial cells [54,55,56].

Chronic exposure to these agents in poorly ventilated kitchens results in cumulative DNA damage and sustained inflammatory signaling through NF-κB and IL-6 activation, promoting epithelial–mesenchymal transition and malignant transformation [57,58]. Epidemiologic studies from Taiwan, China, and Singapore have shown that women with prolonged exposure to cooking fumes without proper ventilation have a 1.5–3.0-fold higher risk of lung adenocarcinoma even in the absence of smoking history [52,59,60].

### 3.2. Environmental Carcinogens and Lung Cancer Risk: Evidence from Epidemiologic and Mechanistic Studies

A range of environmental and lifestyle exposures contribute substantially to lung carcinogenesis beyond active tobacco smoking. These include inorganic arsenic, asbestos, radon (Rn-222), secondhand smoke, ionizing radiation, and chronic alcohol intake, each acting through overlapping mechanisms involving oxidative stress, DNA damage, and chronic inflammation.

Arsenic exposure, mainly through contaminated groundwater and occupational settings, induces oxidative DNA damage, chromosomal instability, and epigenetic silencing of tumor suppressor genes such as *TP53* and *CDKN2A* [61,62,63]. Long-term exposure in regions such as Taiwan and Bangladesh correlates with increased lung cancer incidence among nonsmokers, with a clear dose–response pattern [64].

Asbestos fibers, particularly chrysotile and amphiboles, provoke chronic pulmonary inflammation and physically disrupt mitosis, leading to TP53 and BAP1 mutations [65,66,67]. Combined exposure to asbestos and cigarette smoke amplifies lung cancer risk up to fivefold compared with unexposed nonsmokers [67].

Radon (Rn-222), a radioactive gas derived from uranium decay, represents the second leading cause of lung cancer worldwide, particularly in confined, poorly ventilated spaces [68]. Ionizing radiation from radon decay products induces double-strand DNA breaks and increases the mutation burden in *TP53*, *KRAS*, and *EGFR* [69,70].

Second-hand smoke exposure results in DNA adduct formation and cytokine-driven inflammation in bronchial epithelial cells, with meta-analyses indicating a 20–30% higher lung cancer risk among lifelong nonsmokers [71,72]. Repeated diagnostic imaging involving low-dose X-ray exposure has also been associated with cumulative genomic instability, although the risk remains relatively modest [73]. Alcohol consumption contributes to carcinogenesis through acetaldehyde-induced DNA adducts and CYP2E1-dependent oxidative stress [74,75].

### 3.3. Ambient Fine Particulate Matter (PM_2.5_) Exposure and Lung Cancer Risk

Experimental evidence demonstrates that PM_2.5_ exposure induces oxidative DNA lesions such as 8-OHdG formation, alters DNA methylation, and activates oncogenic pathways including *EGFR*, *KRAS*, and *PI3K*/*AKT* [76,77,78,79]. Chronic exposure drives epithelial–mesenchymal transition, angiogenesis, and immune evasion through upregulation of PD-L1 expression on bronchial epithelial cells [80,81,82].

Epidemiologic studies consistently reveal a dose-dependent association between long-term PM_2.5_ exposure and lung cancer incidence, including among nonsmokers. Global pooled analyses estimate that each 10 μg/m^3^ increase in PM_2.5_ concentration raises lung cancer mortality by 8–14% [83,84]. The effect is most pronounced for adenocarcinoma, reflecting peripheral airway deposition patterns [85]. Molecular epidemiology further shows enrichment of *EGFR*-mutant, *KRAS*-wild-type tumors among individuals exposed to PM_2.5_ [86].

Asian population studies indicate that urban PM_2.5_ concentrations frequently exceed WHO limits (<5 μg/m^3^ annual mean), often reaching 25–50 μg/m^3^. In Taiwan, long-term exposure has been strongly correlated with increased lung adenocarcinoma risk among never-smoking women [87]. These findings reinforce PM_2.5_ as a potent carcinogen independent of tobacco exposure. These observations support an association between environmental exposures and lung cancer risk and histologic patterns in never-smokers, although causal inference remains limited by heterogeneity in exposure assessment [18].

### 3.4. Familial Risk for Lung Cancer

Lung cancer is strongly linked to environmental exposures such as smoking and air pollution, but evidence also shows clear familial clustering [88,89,90]. Individuals with a family history of lung cancer have a two to threefold higher risk of developing the disease, particularly when siblings are affected [91,92]. This elevated risk is consistent across genders, ethnicities, and tumor types [93,94].

Population-based studies from countries such as Sweden and Iceland demonstrate increased risk among first-, second-, and third-degree relatives, suggesting a meaningful genetic contribution [95,96]. Higher risk observed in spouses indicates that shared environmental exposure also plays an important role [92].

Familial susceptibility appears especially important in never-smokers and in women. In high risk regions such as Xuanwei in China, both severe air pollution and inherited factors contribute to the high incidence of lung cancer, particularly among female relatives [97].

Only a limited number of genes have been confirmed in familial lung cancer. Variants in *EGFR*, *RGS17*, and the 15q24 to 25.1 locus are associated with increased susceptibility [98,99,100,101]. Advances in whole-genome and exome sequencing continue to identify new candidate genes and improve understanding of hereditary risk.

### 3.5. Previous Lung Diseases and Lung Cancer

Previous lung diseases (PLD) are important risk factors for lung cancer. In this meta-analysis of 73 studies, asthma, chronic bronchitis, emphysema, pneumonia, tuberculosis, and chronic obstructive pulmonary disease (COPD) were all associated with elevated lung cancer risk, and hay fever was linked to reduced risk. Individuals with COPD or emphysema had at least double the risk, highlighting the need for closer monitoring and consideration for lung cancer screening [102]. Previous lung diseases should be more precisely classified as smoking-related or smoking-independent, as these conditions may differentially influence lung cancer risk in never-smokers. Smoking-related diseases such as idiopathic pulmonary fibrosis [103], chronic obstructive pulmonary disease (COPD) [104,105,106], and emphysema [106] are strongly linked to tobacco exposure. In contrast, smoking-independent conditions, including prior tuberculosis [107], bronchiectasis [108], and chronic inflammatory lung disease [109], have been associated with an increased risk of lung cancer even among never-smokers.

### 3.6. Distribution of Driver Mutations by Smoking Status Across Lung Cancer Subtypes

Driver mutation frequencies stratified by smoking status are shown in Table 1. In small-cell lung cancer (SCLC), TP53 mutations were less frequent in never-smokers than in current/former smokers (59% vs. 85%, *p* < 0.001), whereas *EGFR* (26% vs. 2.6%, *p* < 0.001), *PIK3CA* (15% vs. 3.6%, *p* = 0.023), and *OS9* mutations (5.6% vs. 0%, *p* = 0.009) were more common in never-smokers [110], In squamous cell carcinoma (SCC), *STK11* mutations were observed more frequently in never-smokers than in smokers (50% vs. 7%, *p* = 0.026) [111]. Across two independent SCC cohorts, *EGFR* mutation prevalence was higher in never-smokers than in current/former smokers (13% vs. 3%, *p* < 0.001; and 7.9% vs. 0.4%, *p* < 0.001). *MET* mutations in SCC were also more frequent among never-smokers (9.5% vs. 0.4%, *p* < 0.001) [112,113]. In lung adenocarcinoma (LUAD), *EGFR* mutations were more prevalent in never-smokers than in smokers in both a small cohort (62% vs. 13%, *p* < 0.001) and a large cohort (65% vs. 41%, *p* < 0.001) [114,115] In large-cell carcinoma (LCC), no significant differences were observed between never-smokers and current/former smokers in the frequencies of *EGFR*, *KRAS*, *ALK*, or *PIK3CA* mutations (*p* = 0.45, 0.58, 1.00, and 0.62, respectively) [116]. Never-smokers with lung cancer tend to have longer survival than current or former smokers. LCINS exhibits distinct clinical features, occurs more frequently in women, is often diagnosed at more advanced stages, and is predominantly adenocarcinoma [117,118]. PIK3CA alterations were observed in approximately 20% of EGFR-mutated small-cell lung cancer cases (Table 1) [110], although no statistically supported association with never-smoker status can be concluded from these data. PIK3CA variations, along with other gene alterations, may influence cancer progression and could therefore play a crucial role in determining targeted treatment strategies [119].

### 3.7. Therapeutic Advances in Lung Cancer Among Never-Smokers: Key Clinical Trials and Emerging Strategies (2016–2025)

Table 2 summarizes practice-changing and landmark phase III trials in lung cancer published between 2016 and 2025, with outcomes stratified by smoking status. In a 2016 phase III trial of pembrolizumab monotherapy in metastatic NSCLC with PD-L1 expression ≥ 50%, improvement in progression-free survival (PFS) was observed in the overall population, with no PFS improvement reported among never-smokers, whereas PFS improvement was observed among smokers [120]. In a 2018 phase III trial of pembrolizumab combined with pemetrexed and platinum chemotherapy in metastatic non-squamous NSCLC without sensitizing *EGFR* or *ALK* mutations, improvements in overall survival (OS) and PFS were reported in the overall population, with PFS improvement observed in both never-smokers and smokers [121].

In *EGFR*-mutant advanced NSCLC, a 2018 phase III trial comparing osimertinib with standard *EGFR* tyrosine kinase inhibitors demonstrated improvement in disease-free survival (DFS) in the overall population, as well as in both never-smokers and smokers. Similarly, in the adjuvant setting, a 2020 phase III trial in resected stage IB–IIIA *EGFR*-mutant NSCLC reported DFS improvement with osimertinib in the overall population and in both smoking subgroups [122,123]. In a 2022 phase III neoadjuvant trial in resectable stage IB–IIIA NSCLC, nivolumab plus platinum-based chemotherapy was associated with improvement in OS and PFS in the overall population, with no PFS improvement reported among never-smokers and PFS improvement reported among smokers [124]. The phase III ATLAS trial demonstrated improved progression-free survival with atezolizumab plus bevacizumab and chemotherapy in patients with EGFR- or ALK-rearranged or -translocated NSCLC who had progressed with TKI therapy [125]. Both never-smokers and ex/current smokers derived clinical benefit from the atezolizumab plus bevacizumab, paclitaxel, and carboplatin (ABCP) arm compared to the pemetrexed plus carboplatin or cisplatin (PC) arm, with ex/current smokers showing a stronger and statistically significant benefit [125]. In a 2024 phase III trial in advanced or metastatic NSCLC, datopotamab deruxtecan demonstrated PFS improvement in the overall population, with no PFS improvement observed in never-smokers and PFS improvement observed in smokers [126]. Findings from trials and Table 1 indicate that therapies demonstrating statistically significant improvements in OS, PFS, or DFS were consistently impactful for clinical decision-making, leading to updates in treatment guidelines for both never-smokers and smokers (Table 3).

## 4. Discussion

### 4.1. Summary of Evidence

This review highlights the multifactorial etiology and distinct molecular underpinnings of LCINS, a condition that is increasingly recognized as a unique clinical entity rather than a variant of smoking-related disease. Global tobacco control efforts have successfully reduced smoking prevalence; however, the relative incidence of LCINS has continued to rise, particularly among women and East Asian populations. The findings synthesized from recent epidemiologic and molecular studies suggest that the interplay between environmental carcinogens and inherited or somatic genomic alterations drives tumorigenesis in this subgroup.

Studies from Taiwan and other Asian populations indicate that the National Lung Screening Trial (NLST) smoking-based eligibility criteria have limited applicability in regions with a high prevalence of lung cancer among never-smokers, where cases occur predominantly in women and are strongly associated with family history and environmental risk factors [53]. The expanding use of LDCT screening has increased the detection of early-stage and stage 0 lung cancers, particularly among never-smokers, raising concerns about overdiagnosis and overtreatment, especially for subsolid nodules [127]. Although surgery remains the cornerstone of curative treatment and outcomes in never-smokers are generally favorable, these trends support risk-adapted screening and surgical strategies, including gender- and exposure-based models, chest radiography (CXR) triage, and selective use of sublobar resection or active surveillance, to preserve oncologic benefit while minimizing unnecessary interventions [128].

A meta-analysis showed strong and consistent associations for indoor environmental exposures such as cooking fumes (OR = 3.68, 95% CI: 2.67–5.07), solid fuel use (OR = 5.54, 95% CI: 3.15–9.72), environmental tobacco smoke (ETS) (OR = 1.96, 95% CI: 1.36–2.82), and residential radon (OR = 1.82, 95% CI: 1.31–2.54). Evidence for other exposures such as indoor occupational chemical exposure (formaldehyde, benzene) was more limited (OR = 2.92, 95% CI: 1.71–4.97) or based on few studies [18]. Epidemiologic data consistently implicate environmental exposures, such as cooking oil fumes, fine particulate matter (PM_2.5_), radon, asbestos, and arsenic as important contributors to LCINS. Mechanistically, these agents induce oxidative stress, DNA adduct formation, and chronic airway inflammation, leading to mutations in genes critical for genomic stability and cell proliferation (e.g., *TP53*, *EGFR*, *KRAS*) [129]. Among these, PM_2.5_ exposure represents a particularly potent and modifiable risk factor, exerting carcinogenic effects even at concentrations below World Health Organization thresholds. In Asian urban environments, where PM_2.5_ levels frequently exceed 25–50 μg/m^3^, the attributable burden of LCINS is substantial [130]. These observations emphasize the public health importance of improving indoor ventilation, regulating environmental pollutants, and implementing exposure mitigation policies.

Long-term PM_2.5_ exposure was assessed using a validated satellite-based spatiotemporal model with 1 × 1 km resolution derived from NASA aerosol optical depth data [131], supplemented by Taiwan’s national air quality monitoring network [37]. We further emphasize that future studies in never-smoker lung cancer should adopt cumulative, time-weighted exposure assessments integrating ambient, indoor, and personal exposure data, and link these metrics to molecular endpoints to strengthen causal inference. Future studies in never-smoker lung cancer should move beyond single-exposure models by adopting cumulative, time-weighted exposure assessments that integrate satellite-derived PM_2.5_ estimates with personal wearable monitors, indoor air-quality sensors, and standardized questionnaires on cooking practices and ventilation, while linking longitudinal exposure metrics to molecular endpoints such as DNA adducts and mutational signatures to strengthen causal inference.

LCINS exhibits a mutation profile which is markedly distinct from that of smoking-associated lung cancer. The enrichment of *EGFR*, *PIK3CA*, *OS9*, *MET*, and *STK1* mutations and the lower prevalence of TP53 mutation in never-smokers underscore the biological divergence between these subgroups (Table 1). These differences have profound therapeutic implications. The high frequency of *EGFR*-activating mutations explains the exceptional responsiveness of LCINS patients to tyrosine kinase inhibitors (TKIs) rearrangements, which represent additional actionable targets. Conversely, the lower tumor mutational burden (TMB) characteristic of LCINS may limit the efficacy of immune checkpoint blockade, as neoantigen scarcity reduces immune recognition.

PD-1 inhibitors demonstrated greater efficacy than chemotherapy in patients with NSCLC who had a history of smoking. In contrast, among never-smokers, PD-1 inhibitors did not confer a survival advantage over chemotherapy. These differences in treatment efficacy by smoking status should be considered in future clinical guidelines and therapeutic decision-making [132]. Nicotine increases PD-L1 expression by activating the α7-nAChR/STAT3 signaling pathway. This mechanism may contribute to immune evasion in smoking-related lung cancer and has implications for immunotherapy response [133].

Between 2016 and 2025, therapeutic strategies for lung cancer, particularly among never-smokers, have undergone transformative change. Landmark trials established targeted therapies and immunotherapies as cornerstones of care. The integration of osimertinib in both metastatic and adjuvant settings has improved survival and central nervous system control in *EGFR*-mutant NSCLC have achieved durable intracranial efficacy. Although immune checkpoint inhibitors (e.g., pembrolizumab, atezolizumab) have improved overall survival, their benefit is less pronounced in never-smokers’ tumors. Emerging modalities such as antibody–drug conjugates (e.g., datopotamab deruxtecan, trastuzumab deruxtecan) demonstrate promise by delivering targeted cytotoxicity independent of TMB, potentially bridging therapeutic gaps for never-smokers.

**Figure 3:**
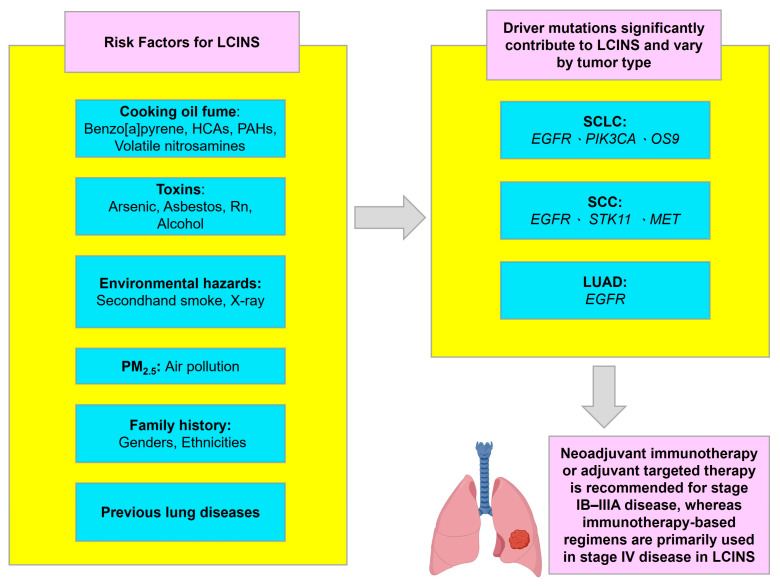
Risk factors, key mutations, and treatment overview for LCINS. LCINS: lung cancer in never-smokers. Icons were created with BioRender.com.

The evidence underscores the critical importance of comprehensive molecular profiling at diagnosis for all NSCLC patients, irrespective of smoking status. Genomic-guided therapy selection not only optimizes treatment outcomes but also minimizes exposure to ineffective regimens. As liquid biopsy and next-generation sequencing become more accessible, early detection of actionable mutations and resistance mechanisms (e.g., *EGFR* T790M, *MET* amplification) will further enhance personalized management. In parallel, preventive strategies, reducing PM_2.5_ exposure, promoting indoor air purification, and enforcing occupational safety should be prioritized (Figure 3). Future research should also explore gene–environment interactions, the role of epigenetic and microbiome alterations, and the potential of combination immunotherapy strategies to overcome resistance in LCINS. Multi-omics approaches integrating germline genomics, somatic mutation profiling, and epigenetic analyses are required to disentangle these contributions.

Smoking status should be treated as a mandatory stratification factor or prespecified subgroup in lung cancer clinical trials. Evidence indicates that treatment efficacy, particularly for immunotherapy and biomarker-driven therapies, differs substantially between never-smokers and smokers, and such stratification allows for accurate interpretation of outcomes and optimization of trial design (Table 2). Murphy et al. highlight that molecularly targeted therapies remain the cornerstone of treatment, delivering median survivals exceeding 3–5 years in advanced disease [3]. Single-agent immune checkpoint inhibitors provide limited benefit in EGFR- or ALK-driven tumors in both smokers and never-smokers [134,135]. For lung cancers without actionable genomic alterations amenable to targeted therapy, chemotherapy combined with immunotherapy can be considered [136]. Future therapeutic strategies may require combination approaches that enhance tumor immunogenicity or target metabolic and stromal dependencies unique to never-smoker lung cancer.

Lung cancer treatment depends on stage, performance status, and tumor molecular features, and may, in the future, also incorporate smoking history, typically combining surgery, radiotherapy, and systemic therapy. As molecular profiling becomes increasingly accessible, precision medicine may complement or partially replace traditional risk factors in guiding screening and treatment decisions for never-smokers. Next-generation sequencing (NGS) is recommended for Stage Ib–IIIa tumors to identify actionable alterations treatable with tyrosine kinase inhibitors (TKIs), which can extend median survival in advanced NSCLC to 3–5 years compared with 1–2 years in the absence of such alterations [3]. In addition, biomarker-based early detection approaches, including blood-based multi-cancer early detection tests and proteomic analyses, are under investigation to enable earlier and more curable lung cancer detection. Lung cancer screening and prevention for never-smokers should adopt risk-adapted strategies that reflect the epidemiology of high-risk Asian populations, particularly women. Screening programs may prioritize individuals with family history, environmental exposures (e.g., PM_2.5_, indoor cooking fumes, radon), and relevant demographic factors, rather than smoking history alone. Prevention efforts should emphasize air quality regulation, indoor ventilation improvement, and exposure reduction, complemented by prospective validation of biomarker-based early detection tools to improve benefit–harm balance and minimize overdiagnosis.

### 4.2. Limitations

This review has several limitations. First, as a narrative rather than a systematic review, selection bias may exist in the choice of included studies and clinical trials. Second, the synthesis primarily focuses on publications from 2016 to 2025, with earlier seminal work referenced selectively, which may limit the historical completeness of the discussion. Third, substantial heterogeneity exists among environmental exposure studies, particularly in methodology, exposure quantification, and regional characteristics, complicating direct comparisons and precluding meta-analytic interpretation. Fourth, many pivotal clinical trials included heterogeneous populations, and subgroup analyses specifically addressing never-smokers were often limited, restricting the generalizability of findings to this population. Finally, some data from 2024 to 2025, particularly those involving antibody–drug conjugates and combination immunotherapy, remain preliminary, with long-term survival outcomes still pending. Future research integrating large scale multi-omics approaches, prospective environmental exposure quantification, and global cohort analyses will be essential to clarify the pathobiology of LCINS and to refine precision prevention and therapeutic strategies. Associations between environmental exposures may be influenced by confounders, including prior smoking history, sex, socioeconomic status, and co-exposures. Exposure measurement is often based on retrospective self-report, household surveys, or indirect estimation (e.g., cooking methods or fuel type), which may introduce recall or misclassification bias. Additionally, smoking history is often not included in cancer registries, databases, and clinical trials, limiting precise estimates of lung cancer incidence in never-smokers and the assessment of treatment outcomes by smoking status. Accurate quantification of environmental exposures, such as air pollution, remains challenging and may lead to exposure misclassification. In addition, the quality of the evidence included was not formally assessed, and relevant studies may have been missed despite a comprehensive search strategy.

## 5. Conclusions

LCINS constitutes a biologically distinct entity driven by complex interactions between environmental carcinogens and specific genomic alterations. The predominance of EGFR mutation and the relative absence of smoking-related mutational signatures distinguish LCINS from conventional lung cancer. Advances in molecularly targeted therapy and immunotherapy have substantially improved survival, but disparities remain due to lower TMB and resistance evolution. Prevention through environmental control, early molecular diagnosis, and precision therapy integration are key to reducing the global burden of LCINS. The continued refinement of biomarker-driven treatment algorithms and the development of novel therapeutic modalities such as ADCs and bispecific antibodies hold promise for further improving outcomes in this growing patient population.

## 6. Clinical Implications

LCINS constitutes a distinct clinical and molecular entity that requires risk-adapted strategies for prevention, screening, and treatment. Smoking history alone is insufficient for risk stratification in this population; environmental exposures (e.g., PM_2.5_, indoor combustion, and radon), family history, and molecular susceptibility should be incorporated into screening frameworks, particularly in high-incidence Asian populations. Comprehensive molecular profiling at diagnosis is essential, as genotype-matched targeted therapies remain the cornerstone of treatment and are associated with superior clinical outcomes compared with immunotherapy alone. Given the generally low tumor mutational burden and limited benefit from immune checkpoint inhibitors in never-smokers, therapeutic decision-making should prioritize targeted agents and emerging modalities such as antibody–drug conjugates. Integration of environmental exposure mitigation, precision diagnostics, and smoking status-stratified clinical trial design is critical for improving outcomes and reducing the growing global burden of LCINS.

## Figures and Tables

**Table 1:** Driver mutations identified in SCLC, LUAD, and SCC, and LCC, stratified by smoking status.

	Gene	Never Smoker	Current/Former Smoker	*p*-Value
SCLC [110]	*TP53*	32/54 (59%)	518/608 (85%)	<0.001
	*EGFR*	14/54 (26%)	16/608 (2.6%)	<0.001
	*PIK3CA*	8/54 (15%)	22/608 (3.6%)	0.023
	*OS9*	3/54 (5.6%)	0/608 (0%)	0.009
SCC [111]	*STK11*	6/12 (50%)	1/14 (7%)	0.026
SCC [112]	*EGFR*	10/76 (13%)	12/353 (3%)	<0.001
SCC [113]	*EGFR*	5/63 (7.9%)	12/2823 (0.4%)	<0.001
	*MET*	6/63 (9.5%)	10/2816 (0.4%)	<0.001
LUAD [114]	*EGFR*	15/24 (62%)	5/38 (13%)	<0.001
LUAD [115]	*EGFR*	5702/8150 (65%)	1477/3528 (41%)	<0.001
LCC [116]	*EGFR*	5/98 (5%)	2/94 (2%)	0.45
	*KRAS*	6/98 (6%)	9/94 (9%)	0.58
	*ALK*	0/98 (0%)	1/94 (1%)	1.00
	*PIK3CA*	3/98 (3%)	1/94 (1%)	0.62

Counts and percentages are reported, *p*-value for difference in proportions was calculated using the chi-squared test. SCLC, Small-cell lung cancer. LUAD, Lung Adenocarcinoma. SCC, Squamous Cell Carcinoma. LCC, Large-cell carcinoma.

**Table 2:** Practice-Changing and Landmark Trials in Lung Cancer (2016–2025): Comparative Outcomes in Never-Smokers and Smokers.

Trial Phase (Year)	Patients	Intervention	Intervention Group	Control Group	Overall Outcomes HR (95% CI)	Outcome in Never-Smokers HR (95% CI)	Outcome in Smokers HR (95% CI)
Phase III (2016) [120]	Metastatic NSCLC, PD-L1 ≥ 50%	Pembrolizumab: PD-1 antibody	Pembrolizumab	Platinum-based chemotherapy	PFS: 0.50 (0.37–0.68)	PFS: 0.90 (0.11–7.59)	PFS: Current Smokers: 0.68 (0.36–1.31) Former Smokers: 0.47 (0.33–0.67)
Phase III (2018) [121]	Metastatic non-squamous NSCLC, without sensitizing EGFR or ALK mutations	Pembrolizumab: PD-1 antibody	Pembrolizumab + pemetrexed + platinum	Placebo + Pemetrexed + platinum	OS: 0.49 (0.38–0.64) PFS: 0.52 (0.43–0.64)	OS: 0.23 (0.10–0.54) PFS: 0.43 (0.23–0.81)	OS: 0.54 (0.41–0.71) PFS: 0.54 (0.43–0.66)
Phase III (2018) [122]	EGFR-mutant advanced NSCLC	Osimertinib: EGFR-TKI	Osimertinib	Standard EGFR-TKI (gefitinib or erlotinib)	PFS: 0.46 (0.37–0.57)	PFS: 0.45 (0.34–0.59)	PFS: 0.48 (0.34–0.68)
Phase III (2020) [123]	Resected stage IB–IIIA EGFR-mutant NSCLC (adjuvant)	Osimertinib: EGFR-TKI	Osimertinib	Placebo	DFS: 0.20 (0.15–0.27)	DFS: 0.23 (0.15–0.34)	DFS: 0.10 (0.04–0.22)
Phase III (2022) [124]	Stage IB–IIIA resectable NSCLC (Neoadjuvant) and ALK translocations or EGFR mutations were excluded.	Nivolumab: PD-1 antibody	Nivolumab plus platinum-based chemotherapy	Platinum- based chemotherapy	EFS: 0.63 (0.45–0.87) pCR: 21.8 (15.2–28.7)	EFS: 0.33 (0.13–0.87) pCR: 10.5 (−7.3–31.4)	EFS: 0.68 (0.48–0.96) pCR: 23.1 (15.9–30.5)
Phase III (2023) [125]	Stage IV EGFR-mutated or ALK- rearranged or translocated NSCLC	Atezolizumab: PD-L1 antibody Bevacizumab: VEGF antibody	Atezolizumab plus bevacizumab, paclitaxel and carboplatin	Pemetrexed plus carboplatin or cisplatin	PFS: 0.63 (0.45–0.86)	PFS 0.67 (0.45–1.01)	PFS 0.53 (0.31–0.93)
Phase III (2024) [126]	Advanced/metastatic NSCLC	Datopotamab-deruxtecan: A TROP2-directed antibody–drug conjugate	Datopotamab-deruxtecan	Docetaxel	PFS: 0.75 (0.62–0.91) OS: 0.94 (0.78–1.14)	PFS: 0.67 (95% confidence interval crossed unity (HR = 1.0)) OS: 1.22 (95% confidence interval crossed unity (HR = 1.0))	PFS: 0.77 OS: 0.88 (95% confidence interval crossed unity (HR = 1.0))

NSCLC: Non-Small-cell lung Cancer. OS, Overall Survival; PFS, Progression-Free Survival; DFS, Disease-Free Survival; EFS, Event-Free Survival. pCR: Pathological Complete Response. HR: Hazard Ratio.

**Table 3:** Practice-Changing and Landmark Trials in Lung Cancer (2016–2025): Overall Comparison Summary.

Feature	LCINS	Current/Former Smoker
Common Genetic Drivers	*EGFR*, *PIK3CA*, *OS9*, *MET*, and *STK11*	*TP53*
Dominant Therapy Type	Adjuvant targeted therapy (stages IB–IIIA)	Neoadjuvant immunotherapy (stages IB–IIIA)
TMB	Lower	Higher
ICI Response Rate	Lower	Higher
Targeted Therapy Response	Higher	Lower
ADC Efficacy	Lower	Higher
Outcome	Excellent with genotype-matched therapy	Improved with ICI and chemo-ICI combinations

TMB: Tumor Mutational Burden. ICI, Immune Checkpoint Inhibitor; ADC, Antibody–Drug Conjugate.

## Data Availability

No new data were created or analyzed in this study.

## Abbreviations

LCINS	Lung cancer in never-smokers
WHO	World Health Organization
IHC	Immunohistochemistry
NSCLC	Non-Small-cell lung Cancer
LUAD	Lung Adenocarcinoma
SCC	Squamous Cell Carcinoma
LCC	Large-cell carcinoma
SCLC	Small-cell lung cancer
OS	Overall survival
PFS	Progression-free survival
pCR	Pathologic complete response
CXR	Chest X-ray
FDG-PET/CT	18F-fluorodeoxyglucose positron emission tomography/computed tomography
EBUS-TBNA	Endobronchial ultrasound-guided transbronchial needle aspiration
NGS	Next generation sequencing
PCR	Polymerase chain reaction
PAHs	Polycyclic aromatic hydrocarbons
HCAs	Heterocyclic amines
ICIs	Checkpoint inhibitors
TMB	Tumor mutational burden
ADC	Antibody–Drug Conjugate

## References

[1] Barta J.A., Powell C.A., Wisnivesky J.P. (2019). Global Epidemiology of Lung Cancer. Ann. Glob. Health.

[2] LoPiccolo J., Gusev A., Christiani D.C., Janne P.A. (2024). Lung cancer in patients who have never smoked—An emerging disease. Nat. Rev. Clin. Oncol..

[3] Murphy C., Pandya T., Swanton C., Solomon B.J. (2025). Lung Cancer in Nonsmoking Individuals: A Review. JAMA.

[4] Kusnierczyk P. (2023). Genetic differences between smokers and never-smokers with lung cancer. Front. Immunol..

[5] Islami F., Torre L.A., Jemal A. (2015). Global trends of lung cancer mortality and smoking prevalence. Transl. Lung Cancer Res..

[6] Cao W., You Z., Wang Z., Liang Z., Li H., Chang Z., Chen Y., Dong G., Cheng Z.J., Sun B. (2025). Risk factors behind the global lung cancer burden: A pan-database exploration. Transl. Lung Cancer Res..

[7] Shirgaonkar R., Mohapatra P.R., Panigrahi M.K., Mishra P., Bhuniya S., Sarkar S., Girija A., Shaik A., Mohanty S., Moorthy A. (2024). Evaluation of Risk Factors for Lung Cancer Among Never Smokers and Their Association With Common Driver Mutations. Cureus.

[8] Filho A.M., Laversanne M., Ferlay J., Colombet M., Pineros M., Znaor A., Parkin D.M., Soerjomataram I., Bray F. (2025). The GLOBOCAN 2022 cancer estimates: Data sources, methods, and a snapshot of the cancer burden worldwide. Int. J. Cancer.

[9] Lu T., Yang X., Huang Y., Zhao M., Li M., Ma K., Yin J., Zhan C., Wang Q. (2019). Trends in the incidence, treatment, and survival of patients with lung cancer in the last four decades. Cancer Manag. Res..

[10] Siegel R.L., Miller K.D., Fuchs H.E., Jemal A. (2022). Cancer statistics, 2022. CA Cancer J. Clin..

[11] Rodak O., Peris-Diaz M.D., Olbromski M., Podhorska-Okolow M., Dziegiel P. (2021). Current Landscape of Non-Small Cell Lung Cancer: Epidemiology, Histological Classification, Targeted Therapies, and Immunotherapy. Cancers.

[12] Tai Q., Zhang L., Hu X. (2020). Clinical characteristics and treatments of large cell lung carcinoma: A retrospective study using SEER data. Transl. Cancer Res..

[13] Dingemans A.C., Fruh M., Ardizzoni A., Besse B., Faivre-Finn C., Hendriks L.E., Lantuejoul S., Peters S., Reguart N., Rudin C.M. (2021). Small-cell lung cancer: ESMO Clinical Practice Guidelines for diagnosis, treatment and follow-up(☆). Ann. Oncol..

[14] Remon J., Soria J.C., Peters S., on behalf of the ESMO Guidelines Committee (2021). Early and locally advanced non-small-cell lung cancer: An update of the ESMO Clinical Practice Guidelines focusing on diagnosis, staging, systemic and local therapy. Ann. Oncol..

[15] Riely G.J., Wood D.E., Ettinger D.S., Aisner D.L., Akerley W., Bauman J.R., Bharat A., Bruno D.S., Chang J.Y., Chirieac L.R. (2024). Non-Small Cell Lung Cancer, Version 4.2024, NCCN Clinical Practice Guidelines in Oncology. J. Natl. Compr. Cancer Netw..

[16] Chang G.C., Chiu C.H., Yu C.J., Chang Y.C., Chang Y.H., Hsu K.H., Wu Y.C., Chen C.Y., Hsu H.H., Wu M.T. (2024). Low-dose CT screening among never-smokers with or without a family history of lung cancer in Taiwan: A prospective cohort study. Lancet Respir. Med..

[17] Cheng E.S., Weber M., Steinberg J., Yu X.Q. (2021). Lung cancer risk in never-smokers: An overview of environmental and genetic factors. Chin. J. Cancer Res..

[18] Hong Y., Jang K.S., Xie H. (2025). Meta-analysis of the association between indoor environmental pollution and lung cancer risk in never-smokers. Am. J. Transl. Res..

[19] An L., Wang Y., Wu G., Wang Z., Shi Z., Liu C., Wang C., Yi M., Niu C., Duan S. (2023). Defining the sensitivity landscape of EGFR variants to tyrosine kinase inhibitors. Transl. Res..

[20] Wong D.W., Leung E.L., So K.K., Tam I.Y., Sihoe A.D., Cheng L.C., Ho K.K., Au J.S., Chung L.P., Pik Wong M. (2009). The EML4-ALK fusion gene is involved in various histologic types of lung cancers from nonsmokers with wild-type EGFR and KRAS. Cancer.

[21] Lin C., Wang S., Xie W., Chang J., Gan Y. (2015). The RET fusion gene and its correlation with demographic and clinicopathological features of non-small cell lung cancer: A meta-analysis. Cancer Biol. Ther..

[22] Hung R.J., Spitz M.R., Houlston R.S., Schwartz A.G., Field J.K., Ying J., Li Y., Han Y., Ji X., Chen W. (2019). Lung Cancer Risk in Never-Smokers of European Descent is Associated With Genetic Variation in the 5(p)15.33 TERT-CLPTM1Ll Region. J. Thorac. Oncol..

[23] Zhou F., Zhou C. (2018). Lung cancer in never smokers-the East Asian experience. Transl. Lung Cancer Res..

[24] Devarakonda S., Li Y., Martins Rodrigues F., Sankararaman S., Kadara H., Goparaju C., Lanc I., Pepin K., Waqar S.N., Morgensztern D. (2021). Genomic Profiling of Lung Adenocarcinoma in Never-Smokers. J. Clin. Oncol..

[25] Pham D., Kris M.G., Riely G.J., Sarkaria I.S., McDonough T., Chuai S., Venkatraman E.S., Miller V.A., Ladanyi M., Pao W. (2006). Use of cigarette-smoking history to estimate the likelihood of mutations in epidermal growth factor receptor gene exons 19 and 21 in lung adenocarcinomas. J. Clin. Oncol..

[26] Gitlitz B.J., Novello S., Vavala T., Bittoni M., Sable-Hunt A., Pavlick D., Hsu R., Park S.L., Chen R., Cooke M. (2021). The Genomics of Young Lung Cancer: Comprehensive Tissue Genomic Analysis in Patients Under 40 With Lung Cancer. JTO Clin. Res. Rep..

[27] Guan Y., Song Z., Li Y., Guo H., Shi J., Zhang X., Yao M. (2019). Effectiveness of EGFR-TKIs in a Patient with Lung Adenocarcinoma Harboring an EGFR-RAD51 Fusion. Oncologist.

[28] Yamaguchi N., Vanderlaan P.A., Folch E., Boucher D.H., Canepa H.M., Kent M.S., Gangadharan S.P., Majid A., Kocher O.N., Goldstein M.A. (2013). Smoking status and self-reported race affect the frequency of clinically relevant oncogenic alterations in non-small-cell lung cancers at a United States-based academic medical practice. Lung Cancer.

[29] Onozato R., Kosaka T., Kuwano H., Sekido Y., Yatabe Y., Mitsudomi T. (2009). Activation of MET by gene amplification or by splice mutations deleting the juxtamembrane domain in primary resected lung cancers. J. Thorac. Oncol..

[30] Dogan S., Shen R., Ang D.C., Johnson M.L., D’Angelo S.P., Paik P.K., Brzostowski E.B., Riely G.J., Kris M.G., Zakowski M.F. (2012). Molecular epidemiology of EGFR and KRAS mutations in 3,026 lung adenocarcinomas: Higher susceptibility of women to smoking-related KRAS-mutant cancers. Clin. Cancer Res..

[31] Ricciuti B., Alessi J.V., Elkrief A., Wang X., Cortellini A., Li Y.Y., Vaz V.R., Gupta H., Pecci F., Barrichello A. (2022). Dissecting the clinicopathologic, genomic, and immunophenotypic correlates of KRAS^G12D^-mutated non-small-cell lung cancer. Ann. Oncol..

[32] Awad M.M., Oxnard G.R., Jackman D.M., Savukoski D.O., Hall D., Shivdasani P., Heng J.C., Dahlberg S.E., Janne P.A., Verma S. (2016). MET Exon 14 Mutations in Non-Small-Cell Lung Cancer Are Associated with Advanced Age and Stage-Dependent MET Genomic Amplification and c-Met Overexpression. J. Clin. Oncol..

[33] Plenker D., Bertrand M., de Langen A.J., Riedel R., Lorenz C., Scheel A.H., Muller J., Bragelmann J., Dassler-Plenker J., Kobe C. (2018). Structural Alterations of MET Trigger Response to MET Kinase Inhibition in Lung Adenocarcinoma Patients. Clin. Cancer Res..

[34] Cho J.H., Ku B.M., Sun J.M., Lee S.H., Ahn J.S., Park K., Ahn M.J. (2018). KIF5B-MET Gene Rearrangement with Robust Antitumor Activity in Response to Crizotinib in Lung Adenocarcinoma. J. Thorac. Oncol..

[35] Liu L.F., Deng J.Y., Lizaso A., Lin J., Sun S. (2022). Effective response to crizotinib of concurrent KIF5B-MET and MET-CDR2-rearranged non-small cell lung cancer: A case report. World J. Clin. Cases.

[36] Arcila M.E., Chaft J.E., Nafa K., Roy-Chowdhuri S., Lau C., Zaidinski M., Paik P.K., Zakowski M.F., Kris M.G., Ladanyi M. (2012). Prevalence, clinicopathologic associations, and molecular spectrum of ERBB2 (HER2) tyrosine kinase mutations in lung adenocarcinomas. Clin. Cancer Res..

[37] Ho C.C., Chen L.J., Hwang J.S. (2020). Estimating ground-level PM_2.5_ levels in Taiwan using data from air quality monitoring stations and high coverage of microsensors. Environ. Pollut..

[38] Li B.T., Smit E.F., Goto Y., Nakagawa K., Udagawa H., Mazieres J., Nagasaka M., Bazhenova L., Saltos A.N., Felip E. (2022). Trastuzumab Deruxtecan in HER2-Mutant Non-Small-Cell Lung Cancer. N. Engl. J. Med..

[39] Goto K., Goto Y., Kubo T., Ninomiya K., Kim S.W., Planchard D., Ahn M.J., Smit E.F., de Langen A.J., Perol M. (2023). Trastuzumab Deruxtecan in Patients with HER2-Mutant Metastatic Non-Small-Cell Lung Cancer: Primary Results From the Randomized, Phase II DESTINY-Lung02 Trial. J. Clin. Oncol..

[40] Calles A., Liao X., Sholl L.M., Rodig S.J., Freeman G.J., Butaney M., Lydon C., Dahlberg S.E., Hodi F.S., Oxnard G.R. (2015). Expression of PD-1 and Its Ligands, PD-L1 and PD-L2, in Smokers and Never Smokers with KRAS-Mutant Lung Cancer. J. Thorac. Oncol..

[41] Tseng J.S., Yang T.Y., Wu C.Y., Ku W.H., Chen K.C., Hsu K.H., Huang Y.H., Su K.Y., Yu S.L., Chang G.C. (2018). Characteristics and Predictive Value of PD-L1 Status in Real-World Non-Small Cell Lung Cancer Patients. J. Immunother..

[42] Otano I., Ucero A.C., Zugazagoitia J., Paz-Ares L. (2023). At the crossroads of immunotherapy for oncogene-addicted subsets of NSCLC. Nat. Rev. Clin. Oncol..

[43] Sabari J.K., Leonardi G.C., Shu C.A., Umeton R., Montecalvo J., Ni A., Chen R., Dienstag J., Mrad C., Bergagnini I. (2018). PD-L1 expression, tumor mutational burden, and response to immunotherapy in patients with MET exon 14 altered lung cancers. Ann. Oncol..

[44] Negrao M.V., Skoulidis F., Montesion M., Schulze K., Bara I., Shen V., Xu H., Hu S., Sui D., Elamin Y.Y. (2021). Oncogene-specific differences in tumor mutational burden, PD-L1 expression, and outcomes from immunotherapy in non-small cell lung cancer. J. Immunother. Cancer.

[45] Chang G.C., Yang T.Y., Chen K.C., Hsu K.H., Huang Y.H., Su K.Y., Yu S.L., Tseng J.S. (2020). ALK variants, PD-L1 expression, and their association with outcomes in ALK-positive NSCLC patients. Sci. Rep..

[46] Lee J., Park C.K., Yoon H.K., Sa Y.J., Woo I.S., Kim H.R., Kim S.Y., Kim T.J. (2019). PD-L1 expression in ROS1-rearranged non-small cell lung cancer: A study using simultaneous genotypic screening of EGFR, ALK, and ROS1. Thorac. Cancer.

[47] Evans M., O’Sullivan B., Hughes F., Mullis T., Smith M., Trim N., Taniere P. (2020). The Clinicopathological and Molecular Associations of PD-L1 Expression in Non-small Cell Lung Cancer: Analysis of a Series of 10,005 Cases Tested with the 22C3 Assay. Pathol. Oncol. Res..

[48] Liu Z., Muehlbauer K.R., Schmeiser H.H., Hergenhahn M., Belharazem D., Hollstein M.C. (2005). p53 mutations in benzo(a)pyrene-exposed human p53 knock-in murine fibroblasts correlate with p53 mutations in human lung tumors. Cancer Res..

[49] Zhang X., Leng S., Qiu M., Ding Y., Zhao L., Ma N., Sun Y., Zheng Z., Wang S., Li Y. (2023). Chemical fingerprints and implicated cancer risks of Polycyclic aromatic hydrocarbons (PAHs) from fine particulate matter deposited in human lungs. Environ. Int..

[50] Bukowska B., Mokra K., Michalowicz J. (2022). Benzo[a]pyrene-Environmental Occurrence, Human Exposure, and Mechanisms of Toxicity. Int. J. Mol. Sci..

[51] Sugimura T., Nagao M., Wakabayashi K. (1994). Heterocyclic amines in cooked foods: Candidates for causation of common cancers. J. Natl. Cancer Inst..

[52] Seow A., Poh W.T., Teh M., Eng P., Wang Y.T., Tan W.C., Yu M.C., Lee H.P. (2000). Fumes from meat cooking and lung cancer risk in Chinese women. Cancer Epidemiol. Biomark. Prev..

[53] Wu F.Z., Huang Y.L., Wu C.C., Tang E.K., Chen C.S., Mar G.Y., Yen Y., Wu M.T. (2016). Assessment of Selection Criteria for Low-Dose Lung Screening CT Among Asian Ethnic Groups in Taiwan: From Mass Screening to Specific Risk-Based Screening for Non-Smoker Lung Cancer. Clin. Lung Cancer.

[54] Wu P.F., Chiang T.A., Ko Y.C., Lee H. (1999). Genotoxicity of fumes from heated cooking oils produced in Taiwan. Environ. Res..

[55] Lin S., Wang H., Cai L., Liao L., Su Y., Cai X., Shen M. (2023). Characteristics and health risk assessment of volatile N-nitrosamines in the plasma of adults in Guangdong Province, China. J. Pharm. Biomed. Anal..

[56] Li K., Ricker K., Tsai F.C., Hsieh C.J., Osborne G., Sun M., Marder M.E., Elmore S., Schmitz R., Sandy M.S. (2021). Estimated Cancer Risks Associated with Nitrosamine Contamination in Commonly Used Medications. Int. J. Environ. Res. Public Health.

[57] Liu J., Fu M., Miao J., Sun Y., Zhu R., Liu C., Bi R., Wang S., Cao X. (2022). The toxicity of cooking oil fumes on human bronchial epithelial cells through ROS-mediated MAPK, NF-kappaB signaling pathways and NLRP3 inflammasome. Environ. Toxicol..

[58] Tadokoro T., Wang Y., Barak L.S., Bai Y., Randell S.H., Hogan B.L. (2014). IL-6/STAT3 promotes regeneration of airway ciliated cells from basal stem cells. Proc. Natl. Acad. Sci. USA.

[59] Ko Y.C., Cheng L.S., Lee C.H., Huang J.J., Huang M.S., Kao E.L., Wang H.Z., Lin H.J. (2000). Chinese food cooking and lung cancer in women nonsmokers. Am. J. Epidemiol..

[60] Chen T.Y., Fang Y.H., Chen H.L., Chang C.H., Huang H., Chen Y.S., Liao K.M., Wu H.Y., Chang G.C., Tsai Y.H. (2020). Impact of cooking oil fume exposure and fume extractor use on lung cancer risk in non-smoking Han Chinese women. Sci. Rep..

[61] Wang C.W., Chiou H.C., Chen S.C., Wu D.W., Lin H.H., Chen H.C., Liao W.T., Lin M.H., Hung C.H., Kuo C.H. (2023). Arsenic exposure and lung fibrotic changes-evidence from a longitudinal cohort study and experimental models. Front. Immunol..

[62] George S., Cassidy R.N., Saintilnord W.N., Fondufe-Mittendorf Y. (2023). Epigenomic reprogramming in iAs-mediated carcinogenesis. Adv. Pharmacol..

[63] Issanov A., Adewusi B., Saint-Jacques N., Dummer T.J.B. (2024). Arsenic in drinking water and lung cancer: A systematic review of 35 years of evidence. Toxicol. Appl. Pharmacol..

[64] Hsu K.H., Tsui K.H., Hsu L.I., Chiou H.Y., Chen C.J. (2017). Dose-Response Relationship between Inorganic Arsenic Exposure and Lung Cancer among Arseniasis Residents with Low Methylation Capacity. Cancer Epidemiol. Biomark. Prev..

[65] Kwak K., Kang D., Paek D. (2022). Environmental exposure to asbestos and the risk of lung cancer: A systematic review and meta-analysis. Occup. Environ. Med..

[66] Oehl K., Vrugt B., Wagner U., Kirschner M.B., Meerang M., Weder W., Felley-Bosco E., Wollscheid B., Bankov K., Demes M.C. (2021). Alterations in BAP1 Are Associated with Cisplatin Resistance through Inhibition of Apoptosis in Malignant Pleural Mesothelioma. Clin. Cancer Res..

[67] Klebe S., Leigh J., Henderson D.W., Nurminen M. (2019). Asbestos, Smoking and Lung Cancer: An Update. Int. J. Environ. Res. Public Health.

[68] Darby S., Hill D., Deo H., Auvinen A., Barros-Dios J.M., Baysson H., Bochicchio F., Falk R., Farchi S., Figueiras A. (2006). Residential radon and lung cancer-detailed results of a collaborative analysis of individual data on 7148 persons with lung cancer and 14,208 persons without lung cancer from 13 epidemiologic studies in Europe. Scand. J. Work. Environ. Health.

[69] Lim S.M., Choi J.W., Hong M.H., Jung D., Lee C.Y., Park S.Y., Shim H.S., Sheen S., Kwak K.I., Kang D.R. (2019). Indoor radon exposure increases tumor mutation burden in never-smoker patients with lung adenocarcinoma. Lung Cancer.

[70] Kashkinbayev Y., Kazhiyakhmetova B., Altaeva N., Bakhtin M., Tarlykov P., Saifulina E., Aumalikova M., Ibrayeva D., Bolatov A. (2025). Radon Exposure and Cancer Risk: Assessing Genetic and Protein Markers in Affected Populations. Biology.

[71] Possenti I., Romelli M., Carreras G., Biffi A., Bagnardi V., Specchia C., Gallus S., Lugo A. (2024). Association between second-hand smoke exposure and lung cancer risk in never-smokers: A systematic review and meta-analysis. Eur. Respir. Rev..

[72] Elkefi S., Zeinoun G., Tounsi A., Bruzzese J.M., Lelutiu-Weinberger C., Matthews A.K. (2025). Second-Hand Smoke Exposure and Risk of Lung Cancer Among Nonsmokers in the United States: A Systematic Review and Meta-Analysis. Int. J. Environ. Res. Public Health.

[73] Lin E.C. (2010). Radiation risk from medical imaging. Mayo Clin. Proc..

[74] Bagnardi V., Rota M., Botteri E., Scotti L., Jenab M., Bellocco R., Tramacere I., Pelucchi C., Negri E., La Vecchia C. (2011). Alcohol consumption and lung cancer risk in never smokers: A meta-analysis. Ann. Oncol..

[75] Hirano T. (2011). Alcohol consumption and oxidative DNA damage. Int. J. Environ. Res. Public Health.

[76] Zhai X., Wang J., Sun J., Xin L. (2022). PM_2.5_ induces inflammatory responses via oxidative stress-mediated mitophagy in human bronchial epithelial cells. Toxicol. Res..

[77] Parida T., Daka G., Murapala D., Kolli S.K., Malla R.R., Namuduri S. (2023). PM_2.5_: Epigenetic Alteration in Lung Physiology and Lung Cancer Pathogenesis. Crit. Rev. Oncog..

[78] Chen C.Y., Huang K.Y., Chen C.C., Chang Y.H., Li H.J., Wang T.H., Yang P.C. (2025). The role of PM_2.5_ exposure in lung cancer: Mechanisms, genetic factors, and clinical implications. EMBO Mol. Med..

[79] Meng X., Du W., Sun Z. (2025). Fine particulate matter-induced cardiac developmental toxicity (Review). Exp. Ther. Med..

[80] Xu X., Xie T., Zhou M., Sun Y., Wang F., Tian Y., Chen Z., Xie Y., Wu R., Cen X. (2024). Hsc70 promotes anti-tumor immunity by targeting PD-L1 for lysosomal degradation. Nat. Commun..

[81] Shen C., Liu J., Zhu F., Lei R., Cheng H., Zhang C., Sui X., Ding L., Yang M., Chen H. (2019). The effects of cooking oil fumes-derived PM_2.5_ on blood vessel formation through ROS-mediated NLRP3 inflammasome pathway in human umbilical vein endothelial cells. Ecotoxicol. Environ. Saf..

[82] Zhu F., Cheng H., Lei R., Shen C., Liu J., Hou L., Zhang C., Xu Y., Ding R., Cao J. (2019). Effects of cooking oil fume derived fine particulate matter on blood vessel formation through the VEGF/VEGFR2/MEK1/2/ERK1/2/mTOR pathway in human umbilical vein endothelial cells. Environ. Toxicol. Pharmacol..

[83] Turner M.C., Krewski D., Pope C.A., Chen Y., Gapstur S.M., Thun M.J. (2011). Long-term ambient fine particulate matter air pollution and lung cancer in a large cohort of never-smokers. Am. J. Respir. Crit. Care Med..

[84] Yang X., Zhang T., Zhang X., Chu C., Sang S. (2022). Global burden of lung cancer attributable to ambient fine particulate matter pollution in 204 countries and territories, 1990–2019. Environ Res..

[85] Pope C.A., Burnett R.T., Thun M.J., Calle E.E., Krewski D., Ito K., Thurston G.D. (2002). Lung cancer, cardiopulmonary mortality, and long-term exposure to fine particulate air pollution. JAMA.

[86] Han S.C., Wang G.Z., Zhou G.B. (2023). Air pollution, EGFR mutation, and cancer initiation. Cell Rep. Med..

[87] Lin W.C., Shie R.H., Yuan T.H., Tseng C.H., Chiang C.J., Lee W.C., Chan C.C. (2024). A nationwide case-referent study on elevated risks of adenocarcinoma lung cancer by long-term PM_2.5_ exposure in Taiwan since 1997. Environ. Res..

[88] Yu X.J., Yang M.J., Zhou B., Wang G.Z., Huang Y.C., Wu L.C., Cheng X., Wen Z.S., Huang J.Y., Zhang Y.D. (2015). Characterization of Somatic Mutations in Air Pollution-Related Lung Cancer. EBioMedicine.

[89] Wu A.H., Fontham E.T., Reynolds P., Greenberg R.S., Buffler P., Liff J., Boyd P., Correa P. (1996). Family history of cancer and risk of lung cancer among lifetime nonsmoking women in the United States. Am. J. Epidemiol..

[90] Bromen K., Pohlabeln H., Jahn I., Ahrens W., Jockel K.H. (2000). Aggregation of lung cancer in families: Results from a population-based case-control study in Germany. Am. J. Epidemiol..

[91] Kanwal M., Ding X.J., Cao Y. (2017). Familial risk for lung cancer. Oncol. Lett..

[92] Hemminki K., Dong C., Vaittinen P. (2001). Cancer risks to spouses and offspring in the Family-Cancer Database. Genet. Epidemiol..

[93] Laguna J.C., Garcia-Pardo M., Alessi J., Barrios C., Singh N., Al-Shamsi H.O., Loong H., Ferriol M., Recondo G., Mezquita L. (2024). Geographic differences in lung cancer: Focus on carcinogens, genetic predisposition, and molecular epidemiology. Ther. Adv. Med. Oncol..

[94] Gee K., Yendamuri S. (2024). Lung cancer in females-sex-based differences from males in epidemiology, biology, and outcomes: A narrative review. Transl. Lung Cancer Res..

[95] Lichtenstein P., Holm N.V., Verkasalo P.K., Iliadou A., Kaprio J., Koskenvuo M., Pukkala E., Skytthe A., Hemminki K. (2000). Environmental and heritable factors in the causation of cancer-analyses of cohorts of twins from Sweden, Denmark, and Finland. N. Engl. J. Med..

[96] Jonsson S., Thorsteinsdottir U., Gudbjartsson D.F., Jonsson H.H., Kristjansson K., Arnason S., Gudnason V., Isaksson H.J., Hallgrimsson J., Gulcher J.R. (2004). Familial risk of lung carcinoma in the Icelandic population. JAMA.

[97] Jin Y., Zhou X., He X. (2001). The general measurement of genetic factors on lung cancer in Xuanwei, China. Zhongguo Fei Ai Za Zhi.

[98] Oxnard G.R., Miller V.A., Robson M.E., Azzoli C.G., Pao W., Ladanyi M., Arcila M.E. (2012). Screening for germline EGFR T790M mutations through lung cancer genotyping. J. Thorac. Oncol..

[99] Bell D.W., Gore I., Okimoto R.A., Godin-Heymann N., Sordella R., Mulloy R., Sharma S.V., Brannigan B.W., Mohapatra G., Settleman J. (2005). Inherited susceptibility to lung cancer may be associated with the T790M drug resistance mutation in EGFR. Nat. Genet..

[100] You M., Wang D., Liu P., Vikis H., James M., Lu Y., Wang Y., Wang M., Chen Q., Jia D. (2009). Fine mapping of chromosome 6q23-25 region in familial lung cancer families reveals RGS17 as a likely candidate gene. Clin. Cancer Res..

[101] Spitz M.R., Amos C.I., Dong Q., Lin J., Wu X. (2008). The CHRNA5-A3 region on chromosome 15q24-25.1 is a risk factor both for nicotine dependence and for lung cancer. J. Natl. Cancer Inst..

[102] Ang L., Ghosh P., Seow W.J. (2021). Association between previous lung diseases and lung cancer risk: A systematic review and meta-analysis. Carcinogenesis.

[103] Yoon H.Y., Kim H., Bae Y., Song J.W. (2024). Smoking status and clinical outcome in idiopathic pulmonary fibrosis: A nationwide study. Respir. Res..

[104] Yoshida T., Tuder R.M. (2007). Pathobiology of cigarette smoke-induced chronic obstructive pulmonary disease. Physiol. Rev..

[105] Goldkorn T., Filosto S., Chung S. (2014). Lung injury and lung cancer caused by cigarette smoke-induced oxidative stress: Molecular mechanisms and therapeutic opportunities involving the ceramide-generating machinery and epidermal growth factor receptor. Antioxid. Redox Signal.

[106] Bhatt S.P., Kim Y.I., Harrington K.F., Hokanson J.E., Lutz S.M., Cho M.H., DeMeo D.L., Wells J.M., Make B.J., Rennard S.I. (2018). Smoking duration alone provides stronger risk estimates of chronic obstructive pulmonary disease than pack-years. Thorax.

[107] Qin Y., Chen Y., Chen J., Xu K., Xu F., Shi J. (2022). The relationship between previous pulmonary tuberculosis and risk of lung cancer in the future. Infect. Agents Cancer.

[108] Choi H., Park H.Y., Han K., Yoo J., Shin S.H., Yang B., Kim Y., Park T.S., Park D.W., Moon J.Y. (2022). Non-Cystic Fibrosis Bronchiectasis Increases the Risk of Lung Cancer Independent of Smoking Status. Ann. Am. Thorac. Soc..

[109] D’Arcy M.E., Pfeiffer R.M., Bradley M.C., Hoang P.H., Tran T.V., McElderry J.P., Li M., Kebede M., DellaValle C.T., Rivas S. (2025). Inflammatory diseases and risk of lung cancer among individuals who have never smoked. Nat. Commun..

[110] Oh M.S., Garon E.B., Lisberg A.E., Cummings A.L., Barrett A., Ashok A., Mauer E., Yilma B., Goldman J.W. (2025). Brief Report: The Genomic Landscape of Small Cell Lung Cancer in Never-Smoking Patients. Clin. Lung Cancer.

[111] Lee H.Y., Lee S.H., Won J.K., Lee D.S., Kwon N.J., Choi S.M., Lee J., Lee C.H., Lee S.M., Yim J.J. (2017). Analysis of Fifty Hotspot Mutations of Lung Squamous Cell Carcinoma in Never-smokers. J. Korean Med. Sci..

[112] Huang Y., Wang R., Pan Y., Zhang Y., Li H., Cheng C., Zheng D., Zheng S., Li Y., Shen X. (2016). Clinical and genetic features of lung squamous cell cancer in never-smokers. Oncotarget.

[113] Reuss J.E., Zaemes J., Gandhi N., Walker P., Patel S.P., Xiu J., Aggarwal C., Vanderwalde A., Ramalingam S.S., Halmos B. (2025). Comprehensive molecular profiling of squamous non-small cell lung cancer reveals high incidence of actionable genomic alterations among patients with no history of smoking. Lung Cancer.

[114] Zhang T., Hoang P.H., Wong J.Y.Y., Yang K., Chen K., Wong M.P., Vermeulen R.C.H., Huang Y., Chanock S.J., Xuanwei Study Team (2023). Distinct Genomic Landscape of Lung Adenocarcinoma from Household Use of Smoky Coal. Am. J. Respir. Crit. Care Med..

[115] Tseng C.H., Chiang C.J., Tseng J.S., Yang T.Y., Hsu K.H., Chen K.C., Wang C.L., Chen C.Y., Yen S.H., Tsai C.M. (2017). EGFR mutation, smoking, and gender in advanced lung adenocarcinoma. Oncotarget.

[116] Yang J., Li Y., Ma B., Xie H., Chen L., Gao X., He W. (2020). Druggable driver gene alterations in redefined large cell carcinoma in Chinese patients: An observational study. Transl. Cancer Res..

[117] Casal-Mourino A., Valdes L., Barros-Dios J.M., Ruano-Ravina A. (2019). Lung cancer survival among never smokers. Cancer Lett..

[118] Paik P.K., Johnson M.L., D’Angelo S.P., Sima C.S., Ang D., Dogan S., Miller V.A., Ladanyi M., Kris M.G., Riely G.J. (2012). Driver mutations determine survival in smokers and never-smokers with stage IIIB/IV lung adenocarcinomas. Cancer.

[119] Cokpinar S., Erdogdu I.H., Orenay-Boyacioglu S., Boyacioglu O., Kahraman-Cetin N., Meteoglu I. (2024). PIK3CA Mutations and Co-Mutations in Operated Non-Small Cell Lung Carcinoma. J. Clin. Med..

[120] Reck M., Rodriguez-Abreu D., Robinson A.G., Hui R., Csoszi T., Fulop A., Gottfried M., Peled N., Tafreshi A., Cuffe S. (2016). Pembrolizumab versus Chemotherapy for PD-L1-Positive Non-Small-Cell Lung Cancer. N. Engl. J. Med..

[121] Gandhi L., Rodriguez-Abreu D., Gadgeel S., Esteban E., Felip E., De Angelis F., Domine M., Clingan P., Hochmair M.J., Powell S.F. (2018). Pembrolizumab plus Chemotherapy in Metastatic Non-Small-Cell Lung Cancer. N. Engl. J. Med..

[122] Soria J.C., Ohe Y., Vansteenkiste J., Reungwetwattana T., Chewaskulyong B., Lee K.H., Dechaphunkul A., Imamura F., Nogami N., Kurata T. (2018). Osimertinib in Untreated EGFR-Mutated Advanced Non-Small-Cell Lung Cancer. N. Engl. J. Med..

[123] Wu Y.L., Tsuboi M., He J., John T., Grohe C., Majem M., Goldman J.W., Laktionov K., Kim S.W., Kato T. (2020). Osimertinib in Resected EGFR-Mutated Non-Small-Cell Lung Cancer. N. Engl. J. Med..

[124] Forde P.M., Spicer J., Lu S., Provencio M., Mitsudomi T., Awad M.M., Felip E., Broderick S.R., Brahmer J.R., Swanson S.J. (2022). Neoadjuvant Nivolumab plus Chemotherapy in Resectable Lung Cancer. N. Engl. J. Med..

[125] Park S., Kim T.M., Han J.Y., Lee G.W., Shim B.Y., Lee Y.G., Kim S.W., Kim I.H., Lee S., Kim Y.J. (2024). Phase III, Randomized Study of Atezolizumab Plus Bevacizumab and Chemotherapy in Patients With EGFR- or ALK-Mutated Non-Small-Cell Lung Cancer (ATTLAS, KCSG-LU19-04). J. Clin. Oncol..

[126] Ahn M.J., Tanaka K., Paz-Ares L., Cornelissen R., Girard N., Pons-Tostivint E., Vicente Baz D., Sugawara S., Cobo M., Perol M. (2025). Datopotamab Deruxtecan Versus Docetaxel for Previously Treated Advanced or Metastatic Non-Small Cell Lung Cancer: The Randomized, Open-Label Phase III TROPION-Lung01 Study. J. Clin. Oncol..

[127] Hsin-Hung C., En-Kuei T., Yun-Ju W., Fu-Zong W. (2024). Impact of annual trend volume of low-dose computed tomography for lung cancer screening on overdiagnosis, overmanagement, and gender disparities. Cancer Imaging.

[128] Wu Y.J., Hung Y.C., Tang E.K., Wu F.Z. (2026). Practical Strategy to Mitigate Overdiagnosis in Asian Low-dose Computed Tomography Lung Cancer Screening. Acad. Radiol..

[129] Hubaux R., Becker-Santos D.D., Enfield K.S., Lam S., Lam W.L., Martinez V.D. (2012). Arsenic, asbestos and radon: Emerging players in lung tumorigenesis. Environ. Health.

[130] Ku P.W., Steptoe A., Hamer M., Zaninotto P., Stamatakis E., Lin C.H., Yu B., Hvidtfeldt U.A., Lao X.Q., Lin H.H. (2025). Does ambient PM_2.5_ reduce the protective association of leisure-time physical activity with mortality? A systematic review, meta-analysis, and individual-level pooled analysis of cohort studies involving 1.5 million adults. BMC Med..

[131] Zhang Z., Chang L.Y., Lau A.K.H., Chan T.C., Chieh Chuang Y., Chan J., Lin C., Kai Jiang W., Dear K., Zee B.C.Y. (2017). Satellite-based estimates of long-term exposure to fine particulate matter are associated with C-reactive protein in 30 034 Taiwanese adults. Int. J. Epidemiol..

[132] Li B., Huang X., Fu L. (2018). Impact of smoking on efficacy of PD-1/PD-L1 inhibitors in non-small cell lung cancer patients: A meta-analysis. OncoTargets Ther..

[133] Li Y., Wang W., Wang Y., Zhou C., Zou X., Wang Y., Wang N. (2025). Nicotine-induced PD-L1 expression in lung squamous cell carcinoma is mediated by the alpha7-nAChR/STAT3 signaling pathway. Transl. Cancer Res..

[134] Mazieres J., Drilon A., Lusque A., Mhanna L., Cortot A.B., Mezquita L., Thai A.A., Mascaux C., Couraud S., Veillon R. (2019). Immune checkpoint inhibitors for patients with advanced lung cancer and oncogenic driver alterations: Results from the IMMUNOTARGET registry. Ann. Oncol..

[135] Vokes N.I., Pan K., Le X. (2023). Efficacy of immunotherapy in oncogene-driven non-small-cell lung cancer. Ther. Adv. Med. Oncol..

[136] Dai L., Jin B., Liu T., Chen J., Li G., Dang J. (2021). The effect of smoking status on efficacy of immune checkpoint inhibitors in metastatic non-small cell lung cancer: A systematic review and meta-analysis. EClinicalMedicine.

